# Global Approach to Follow-Up of Celiac Disease

**DOI:** 10.3390/foods13101449

**Published:** 2024-05-08

**Authors:** Gesala Perez-Junkera, Lorea Ruiz de Azua, Maialen Vázquez-Polo, Arrate Lasa, María Pilar Fernandez Gil, Itziar Txurruka, Virginia Navarro, Idoia Larretxi

**Affiliations:** 1 GLUTEN3S Research Group, Department of Nutrition and Food Science, University of the Basque Country, 01006 Vitoria-Gasteiz, Spain; gesala.perez@ehu.eus (G.P.-J.); lruizdeazua004@ikasle.ehu.eus (L.R.d.A.); maialen.vazquez@ehu.eus (M.V.-P.); mariadelpilar.fernandez@ehu.eus (M.P.F.G.); itziar.txurruka@ehu.eus (I.T.); virginia.navarros@ehu.eus (V.N.); idoia.larrechi@ehu.eus (I.L.); 2 Children’s National Hospital 111 Michigan Avenue NW, Washington, DC 20010, USA; 3 Bioaraba, Nutrición y Seguridad Alimentaria, 01006 Vitoria-Gasteiz, Spain; 4 Centro Integral de Atención a Mayores San Prudencio, Ayuntamiento de Vitoria-Gasteiz, 01006 Vitoria-Gasteiz, Spain

**Keywords:** celiac disease, gluten-free diet, dietary adherence, biomarker, intestinal damage, follow-up, nutritional balance, quality of life, psychological assistance, social inclusion

## Abstract

Celiac disease, an autoimmune disorder induced by the ingestion of gluten, affects approximately 1.4% of the population. Gluten damages the villi of the small intestine, producing symptoms such as abdominal pain, bloating and a subsequent loss of nutrient absorption, causing destabilization of the nutritional status. Moreover, gluten can trigger extra intestinal symptoms, such as asthma or dermatitis, but also mental disorders such as depression or anxiety. Moreover, people suffering from celiac disease sometimes feel misunderstood by society, mainly due to the lack of knowledge about the disease and the gluten-free diet. Thus, the treatment and follow-up of patients with celiac disease should be approached from different perspectives, such as the following: (1) a clinical perspective: symptomatology and dietary adherence monitorization; (2) nutritional assessment: dietary balance achievement; (3) psychological assistance: mental disorders avoidance; and (4) social inclusion: educating society about celiac disease in order to avoid isolation of those with celiac disease. The aim of this narrative review is to gain deep insight into the different strategies that currently exist in order to work on each of these perspectives and to clarify how the complete approach of celiac disease follow-up should be undertaken so that the optimum quality of life of this collective is reached.

## 1. Introduction

Celiac disease (CD) is a systemic autoimmune disorder induced by the ingestion of gluten in genetically predisposed individuals, which causes a reversible inflammatory process in the mucosa of the small intestine, leading to the loss of the absorptive villi [1,2,3,4,5]. Currently, approximately 1.4% of the global population suffers from this disease [5,6]. Even so, it is estimated that many more people may have the disease but are undiagnosed due to their lack of symptoms.

Acute symptoms of CD are both intestinal and extra intestinal. The most common intestinal ones are abdominal pain or bloating, accompanied by diarrhea, malabsorption and steatorrhea, which, in the case of children, causes consequent weight loss and growth retardation. Affected individuals also suffer from extra gastrointestinal symptoms such as anemia, fatigue, dermatitis herpetiformis, liver disease, or infertility. Moreover, mental disorders such as depression and anxiety are becoming more common among the population with celiac disease [7].

The only treatment for CD is a gluten-free diet (GFD), which, in addition to being nutritionally balanced, must ensure the complete absence of gluten. This is sometimes a very complex and difficult objective to achieve, since there may be foods that, although naturally gluten-free, are contaminated via cross-contact with other foods containing gluten. There are also some incorrect culinary practices that could lead to involuntary gluten ingestion and thus to the presence of symptoms. Therefore, one of the main guidelines when following a GFD is to ensure proper adherence to the diet, which can be currently measured by means of different tools, such as dietary questionnaires, biopsies, blood tests, etc. [3]. Recently, biomarker detection has been proposed as a possible new non-invasive tool for monitoring the disease [4,8].

Achieving nutritional balance during GFD follow-up is also crucial in order to ensure the nutritional status of people with celiac disease. However, several studies in the literature indicate that nutritional deficiencies are common among these people [9,10,11,12]. These deficiencies can occur due to different reasons. On the one hand, if a GFD is not strictly followed and gluten transgressions occur, intestinal damage can be maintained in the long term, and the absorption of nutrients such as iron, calcium, and vitamin B12 become seriously compromised, leading to complications such as osteoporosis and/or anemia [1,2,3,4]. On the other hand, a GFD is characterized as a restrictive diet where several cereals, which are sources of carbohydrates and other micronutrients, need to be eliminated. Finally, specific GFPs have been demonstrated to be nutritionally different from their gluten-containing counterparts, being richer in saturated fats and poorer in fiber, for example [13,14]. All of this evidence has highlighted the risk of dietary imbalance and, thus, the need for nutritional counselling among people with celiac disease on a GFD.

In addition to these difficulties, another important issue is that people with celiac disease feel misunderstood by society, mostly when they must eat outside the home. The lack of knowledge about CD and the presence of gluten in foods among general society makes people with celiac disease feel different from others or, on some occasions, excluded in some situations. Studies performed to measure the quality of life of the population with celiac disease have shown a poor self-reported quality of life among both children and adults with celiac disease, suggesting the need to measure this aspect in CD follow-up visits [15,16,17]. Thus, psychological assessments may be another component to include in the follow-up. At the same time, actions to promote the spreading of knowledge about CD and GFDs in society should be carried out.

The aim of this narrative review is to define each of the perspectives that the global approach to a CD follow-up should include, starting from the clinical perspective, followed by nutritional and psychological assessments, and finishing with social education. Therefore, this review attempts to serve as a “guideline” for the complete follow-up and monitorization of CD.

## 2. Materials and Methods

This article is a narrative review based on a systematic search of some of the literature. The PubMed database was used to conduct a bibliographic search using different combinations of the following terms: celiac disease, intestinal damage, biomarkers, gluten-free diet, nutritional balance, psychological assessment and quality of life. These terms were selected in order to approach pathology monitoring from different perspectives (clinical, nutritional, psychological and social). Inclusion criteria included observational studies, case–control studies, cohort studies and systematic reviews. Articles published mainly in the last twenty years and in journals in the first and second quartile were selected.

Only studies with participants diagnosed with CD and following a GFD, used for adherence measurement, biomarker detection, symptom analysis, nutritional evaluation and quality of life determination (or at least one of these analyses) were included.

Nevertheless, other important studies will be mentioned in this text in order to clarify, explain or justify some of the observations extracted from selected articles, such as articles analyzing GFDs in more detail (nutritional deficiencies, GFP consumption, GFP composition, etc.).

## 3. The Complete Approach to CD Follow-Up

According to the Celiac Disease Foundation or the Society for the Study of Celiac Disease, CD should be recognized as one of the world’s most prevalent, and least diagnosed, genetic autoimmune diseases. Public health efforts could significantly contribute to reducing the burden of celiac disease. In this sense, current public health challenges in celiac disease include the following: (a) from a clinical and nutritional perspective: diagnosis and early identification through serological screening, varied clinical presentations requiring increased awareness and potential misdiagnosis; regarding treatment, need for trained professionals and multidisciplinary care, inadequate healthcare infrastructure, limited availability and nutritional quality of gluten-free products (lack of commercial gluten-free products in developing countries), high costs, reliable gluten-free food production and proper food labeling for gluten content, gluten contamination, promoting adherence to a GFD, a balanced diet; and (b) from a social and psychological perspective: limited social support, social isolation, discrimination, psychological distress, negative impact on the quality of social life for individuals with celiac disease [18,19,20,21,22].

**Figure 1:**
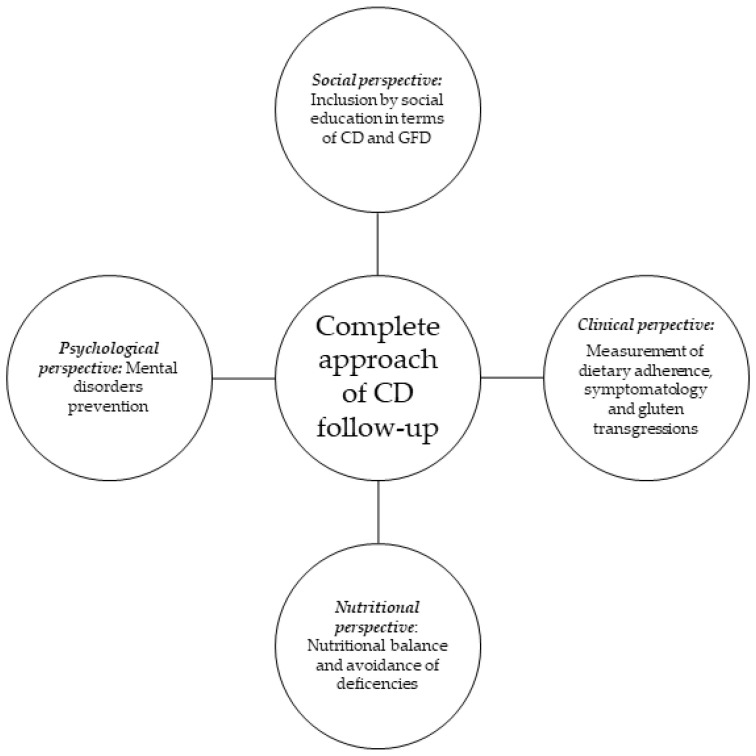
Scheme of how the global approach to CD follow-up should be.

Therefore, a complete approach to CD follow-up should be performed from the previously mentioned four different perspectives: the clinical perspective, focused on the control of dietary adherence and symptomatology; the nutritional assessment for dietary balance acquisition and avoidance of nutrient deficiencies; psychological assistance, attempting to prevent mental disorders; and social inclusion, involving education of the general population regarding GFDs and CD, in order to promote the social integration of the population with celiac disease (Figure 1).

### 3.1. Clinical Perspective: Measurement of Dietary Adherence, Gluten Transgressions and Symptomatology

Strict adherence to a GFD is crucial for the remission of symptoms and intestinal healing. However, the lack of knowledge about potential gluten-containing ingredients in food labels or the risk of cross contamination makes this strict adherence difficult to achieve, especially when eating outside the home. In this sense, several studies have reported that one-third of patients with CD do not fully adhere to a GFD [23,24]. Moreover, the estimated compliance rates reported in patients with CD is highly variable in different studies, ranging from 50% to 90% [25]. This is a very important fact since a small gluten transgression can cause slight or moderate symptomatology but severe intestinal damage that needs long periods of recovering.

**Figure 2:**
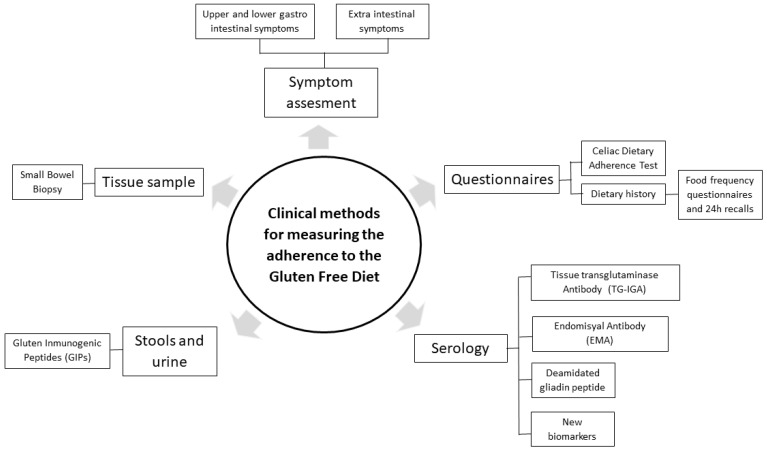
Clinical methods for GFD adherence measurement. Modified from Aljada et al. [3].

Even though it is clear that the control of dietary adherence should be included in the CD follow-up, there is no consensus regarding either the optimal frequency of monitoring compliance or the best tools for assessing it [26]. Along these lines, methods for measuring dietary adherence are being discussed because of their significant limitations and insufficient sensitivity to detect occasional transgressions that may impede full gut mucosa recovery [27,28,29,30,31]. It has been shown that between 36% and 55% of patients who declare themselves as fully adhering to a GFD in questionnaires do not achieve histological remission, probably because of inadvertent lapses or involuntary gluten intakes that cannot be consciously auto-reported [24,32,33,34]. Thus, the combination of questionnaires with other more objective techniques has recently been proposed to measure GFD adherence. Among the objective techniques, traditional ones, such as intestinal biopsy and serological analysis, and new biomarkers currently proposed can be found. The combination of all the data should provide more reliable and complete information about gluten transgressions and adherence to a GFD (Figure 2).

#### 3.1.1. Questionnaires for Dietary Adherence Measurement

Traditionally, different questionnaires for measuring adherence to the GFD have been used due to their ease of application, their low cost and not being invasive [2,3,5,35,36,37,38,39,40]. Among them, there is one regulated survey that has been proposed as a fast tool for the screening of GFD dietary adherence, the Coeliac Disease Adherence Test or CDAT [41]. This questionnaire consists of seven questions, with answers based on a Likert scale, measuring a score of 7 to 12 points (good adherence to the diet), 13 to 17 points (regular adherence), and 18 to 35 points (poor adherence to the diet and need for help). The questionnaire has been used in both children and adults with celiac disease and has been adapted and validated for use in different cultures [39].

The combination of this survey with other dietary questionnaires, such as those measuring 24 h recall and food consumption frequency, could provide deeper insight into the kind of foods that a patient with celiac disease consumes and also into the amount of GFPs in the diet, the portion size and culinary preparation. In this way, the patient’s dietary habits can be known in detail, and possible sources of contamination (even specific products or incorrect handling) can be detected [39].

It is important to emphasize that specific products labeled “gluten-free” (GF) do not ensure the total absence of the protein. Spanish legislation allows for the labelling of a product as GF if it contains less than 20 ppm of gluten (20 mg of gluten per kg of food), which does not guarantee that the product is completely GF [1,2,3,42]. In fact, these products are considered potential vehicles for a small amount of gluten, and therefore, it must be taken into account that the accumulated consumption of these products in just one intake, or in a very short period of time, may result in a recurrence of CD-related symptoms in some people with celiac disease [43]. Moreover, it is remarkable that current food legislation does not oblige companies to declare traces of allergens, including gluten.

Cross-contact between gluten-containing and GF foods is also a matter of concern. Incorrect practices during the culinary preparation can cause these products to be contaminated. This fact highlights the importance of good practices when cooking, such as tools used exclusively for GF foods and the proper cleaning of common machinery for GF and gluten-containing foods (ovens, toasters, frying pans, etc.). Additionally, proper quality systems must be implemented in both the food industries and catering services that produce and serve GF foods and meals so that safety is guaranteed in their products. This information, via home machinery used or restaurants visited, may be directly or indirectly collected through the use of dietary questionnaires.

Finally, a fact worth mentioning is that there are also other non-food products that may be susceptible to being sources of gluten traces because they are often formulated with this protein or with ingredients that contain it. This is the case of some pharmaceuticals or dietary supplements, as well as cosmetic products such as body lotions, shampoos, lipsticks and toothpaste, among others [1,2,3]. Even though it is possible that this information would not be fully obtained via dietary questionnaires, health professionals working with people with celiac disease should also be aware of the use of these products by their patients.

Although important and complete information can be obtained from the above-mentioned questionnaires, it is important to remark that they are not always entirely accurate in measuring dietary adherence, as there might be a potential gap between patients ‘perceptions of a strict GFD and real-life exposure to gluten in a diet or products consumed [25,35,37,38]. Therefore, a combination of these questionnaires with other clinical measurements could probably be more appropriate for obtaining more reliable and objective data.

#### 3.1.2. Questionnaires for Symptom Assessment

The presence of symptoms among patients with celiac disease can be measured through specific questionnaires. Along these lines, a structured Gastrointestinal Symptom Rating Scale (GSRS) questionnaire has been widely used in clinical research [44,45] on celiac disease and other gastrointestinal disorders [46,47]. It consists of a list of gastrointestinal symptoms divided into five sub-dimensions: indigestion, diarrhea, abdominal pain, reflux and constipation. Symptom severity can be determined because answers can vary from mild discomfort to moderate, severe or very severe discomfort.

Regarding extra intestinal symptoms, there is another specific questionnaire that asks about the presence or absence of symptoms such as dermatitis, headache, brain fog, fatigue, numbness of the limbs, joint/muscle pain and fainting. This questionnaire, as well as the one mentioned above, has been widely used in clinical research [48].

Describing the presence of symptoms through the mentioned questionnaires will help health professionals better understand the development of CD. Furthermore, these results, together with data on dietary adherence (obtained through questionnaires or clinical determinations), will give a clearer idea of the pathological status and will be useful for monitoring the disease.

#### 3.1.3. Serological Analysis and Intestinal Biopsy

The detection of serum antibodies such as TTG-IgA and IgA-EMA [3,4] is within the established protocol for diagnosis and follow-up of CD. These parameters, measured throughout a GFD treatment, are indicative of the degree of intestinal recovery. However, several studies have indicated that a normalization of these serological markers does not consistently reflect mucosal healing due to their low sensitivity in detecting villous atrophy [49].

For years, another commonly used tool to determine intestinal mucosal damage has been intestinal biopsy, which has subsequently been evaluated and contrasted with the Marsh classification. There are four degrees of intestinal atrophy according to this classification: Marsh 0, 1, 2, 3a, 3b, 3c and 4, and they are classified according to intraepithelial lymphocyte levels, crypt hyperplasia and villous atrophy [3,50,51]. Although periodic biopsies during a GFD are currently the only reliable tool to demonstrate small intestinal mucosal healing, there is no consensus on the routine use of biopsy in adults, and follow-up biopsy is not performed in children. Moreover, performing this procedure (endoscopic examination) for the demonstration of villous atrophy is invasive and expensive. In fact, current guides of the ESPGHAN emphasize that among children and adolescents, biopsies are not always necessary [52].

Taking this information into account, new procedures that are non-invasive and directly linked to intestinal mucosal damage and/or healing are being studied. New biomarkers have gained importance in this field. Even though more studies are needed to describe all potential biomarkers for celiac disease, it is clear that their detection, in combination with other non-invasive methods such as the dietary questionnaires mentioned above, will provide objective and reliable information about the evolution of the mucosal status [4,8].

#### 3.1.4. Detection of Biomarkers

Biomarker detection found in blood, urine and/or feces has recently been proposed. In this way, dietary monitoring can be supported by objective data to complement the results of questionnaires [4,8]. In this section, these biomarkers are presented, detailing their levels in both CD and during treatment with a GFD, and they are summarized in Table 1.

##### Intestinal Fatty Acid-Binding Proteins (I-FABPs)

Intestinal fatty acid-binding proteins (I-FABPs) are cytosolic proteins found in the mature enterocytes of the intestinal villi in the small intestine, specifically in the jejunum. This area of the intestine is highly affected in the event of intestinal damage, which results in the release of these proteins. Due to their small size and solubility in water, they are quickly released into the bloodstream and then eliminated in the urine through the kidneys. Measurement of I-FABP levels has been studied as being useful in estimating enterocyte damage [4,5,8,53,54].

Several studies have observed that plasma levels of I-FABPs are high in CD patients compared to healthy individuals at diagnosis, indicating mucosal damage [53,54,55,56,57,58,59,60]. In fact, it has been proposed that patients who meet the four criteria for CD diagnosis (clinical picture of CD, tTG-IgA levels above 10 U/mL and IgA-EMA positivity, HLA-DQ2 and/or -DQ8 genotype and intestinal atrophy observed in the biopsy), together with an elevated serum level of I-FABPs, appear to suffer from CD; therefore, a diagnostic biopsy could be omitted in their case [55].

In addition, I-FABP levels may also be useful to monitor the disease from the beginning of a GFD treatment, due to their correlation with intestinal damage and recovery. Along these lines, I-FABP levels in pediatric patients with celiac disease were observed to normalize after 26 weeks on a GFD [55,56,61]. In one of these studies, the authors also observed a reduction in tTG-IgA levels, but they were not normalized even after 6 months on a GFD, which indicates that the decrease in tTG-IgA occurs later than that of I-FABPs. Studies performed in adult patients on a GFD have also described reductions in I-FABPs, but even after one year of treatment, levels of these proteins still differed from those of healthy patients [62]. Thus, the reduction in this biomarker among pediatric patients on a GFD occurs faster than in adults.

##### Cytochrome P450 3A4 (CYP3A4)

CYP3A4 is an enzyme system that is abundantly expressed in the villus tips and, to a lesser extent, in the crypts [4,5,8]. Likewise, the function of enterocytes can be assessed by cytochrome expression and function, which could be an interesting biomarker to evaluate intestinal damage [63].

Clinical studies have described how in intestinal biopsy samples obtained at diagnosis, villous atrophy is observed, and there is no CYP3A4 staining. However, in samples obtained after a GFD treatment, a restructuring of the intestinal villi occurs, and consequently, CYP3A4 staining is positive [64,65].

A non-invasive method for the quantification of CYP3A4 activity consists of the oral administration of simvastatin (SV). SV is a lipid-reducing agent metabolized by cytochrome. Healthy individuals have low levels of SV, and higher concentrations of its metabolites in blood are found; therefore, in patients with enteropathy, SV levels should be high and the levels of its metabolites low [66]. A study by Morón et al. [66] compared the differences in this enzyme system among healthy individuals, patients recently diagnosed with CD and patients with CD treated with a GFD for more than one year. Participants were administered SV, and subsequently, the blood concentration of SV acid, a non-CYP3A4-derived metabolite, was assessed. A significant difference was observed between the values of the untreated patients and the other two groups; the untreated group showed a significantly higher SVeq (SV equivalent) Cmax compared to the treated group and the healthy group. Thus, these results explain how SVeq, which indicates CYP3A4 activity, is a non-invasive marker for detecting the status of the small intestine.

Apart from this investigation, CYP3A4 expression has been also quantified in healthy participants, untreated celiac patients and celiac patients following a GFD, being significantly different among them, lower in untreated patients and higher in treated patients and healthy people [64,66]. Thus, it can be concluded that CYP3A4 expression decreases due to intestinal atrophy caused by gluten ingestion and that, in cases of being treated with a GFD, the mucosa is restructured, and, therefore, CYP3A4 expression increases.

Given these results, it can be concluded that untreated celiac patients with intestinal atrophy have no or low CYP3A4 expression in intestinal samples. The initiation of a GFD improves intestinal damage with villous restructuring and thus increases CYP3A4 expression. The method of studying CYP3A4 by detecting SV in blood is a non-invasive method suitable for studying the state of the intestinal mucosa.

##### Gluten Immunogenic Peptides (GIPs)

GIPs are fragments of gluten proteins resistant to gastrointestinal digestion that trigger immunological reactions in celiac patients, mainly the 33-mer alpha-gliadin peptide. They are excreted in the urine and feces during gluten consumption [1,2,3,4,5,8,40,50,67].

Several studies have assessed the presence of GIPs in urine [68,69,70] and stool samples [71,72,73,74] in celiac patients at diagnosis and after going on a GFD. It has been observed that the presence of GIPs in urine or stool is dependent on gluten intake, showing higher concentrations at diagnosis and lower concentrations during treatment. Nevertheless, even though the number of patients with detectable GIPs decreases during treatment, the peptides continue being detectable in some patients, which demonstrates a lack of compliance with the GFD among them. Therefore, GIP detection has been proposed as a non-invasive biomarker suitable for monitoring dietary adherence.

It is remarkable that GIP values have been correlated to the age of the patients in both children and adults, where the older the age, the higher the amount of GIPs in the feces [71]. This fact has been explained, at least in children, by lower parental control over the followed diet at older ages.

Finally, the relationship between GIP presence in samples and mucosal damage in intestinal biopsies is controversial. While some studies have observed no significant relationship between these two parameters [1,68,75], others have [68]. Thus, more studies are needed to verify these results.

##### Regenerative Gene 1 Alpha (REG1 α)

REG1α is a protein belonging to the regenerative gene family [76]. It is expressed in the pancreas and intestine, and its function is related to tissue regeneration. It is associated with several diseases such as pancreatitis, inflammatory bowel disease or cancer, among others [77]. High levels of REG1α represent the attempt of an organism to regenerate in response to enterocyte damage or death [4,78].

In relation to studies performed in celiac patients, it has been observed that circulating REG1α levels decrease in both pediatric and adult patients after a variable period of time on a GFD, ranging from 6 to 24 months, but no relationship has been found between plasma concentration and the duration of the dietary treatment.

These studies indicate that the recovery of the intestinal mucosa after a GFD treatment is reflected in the decrease in REG1α values. Even so, these data are supported by a single study, and thus, further research is needed to conclude that REG1α is a useful biomarker for monitoring dietary adherence in celiac patients.

##### Citrulline (CIT)

CIT is a non-protein amino acid mainly synthesized by enterocytes, thus representing the healthy function of these cells. It has been established that circulating citrulline levels could be also a marker of intestinal function [4,5,8,79].

Several studies have analyzed the usefulness of the measurement of this protein to observe intestinal damage in CD patients. It has been shown that patients with affected intestines have lower citrulline levels compared to healthy patients and that treatment with a GFD increases this parameter, reaching normal values [79]. However, while normalization is reached after 3 months in pediatric patients, in the case of adult patients, it requires more time [80,81]. Concerning the relation of this protein to intestinal atrophy, even though some discrepancies can be found in the literature, most of the studies describe that the more severe the mucosal damage, the lower the CIT concentration [80,81,82].

The usefulness of this biomarker has also been compared to that of others mentioned above—concretely, to REG1a and I-FABPs. It has demonstrated that in adult patients with CD, CIT may be the most reliable non-invasive biomarker for predicting the presence of villous abnormality because of its high specificity. Moreover, a combination of low CIT and high I-FABP levels has been proposed for higher diagnostic accuracy and for avoiding biopsies [83].

In view of these results, it can be concluded that CIT plasma levels increase with a GFD treatment. There are discrepancies about the correlation between CIT and the degree of villous atrophy; thus, more investigation is needed in this field. Along the same lines, more studies should be focused on determining the duration of GFD needed to normalize serum CIT levels and mucosal recovery. Even so, and considering all the studies globally, we conclude that CIT can alert to enterocyte damage, and it can be a useful tool for monitoring dietary adherence.

##### MicroRNAs (miRNAs)

miRNAs are short non-coding RNA sequences involved in post-transcriptional gene regulation. They play a key role in the pathogenesis of autoimmune and gastrointestinal diseases. Recent studies have investigated their association with several diseases, including CD [2,4,5,40,84].

Different studies have observed decompensated circulating miRNAs among untreated celiac patients, patients treated with GFD and healthy people. Among them, trials performed in pediatric patients revealed that the expression of miR-192-5p, miR-215-5p, miR-125b-5p and miR-21 was increased, and miR-31 was decreased in untreated patients [85,86]. By contrast, in the case of adults, miR-155, miR-21, and miR-125 were elevated, but no significant differences were found between patients on a GFD and those with a GCD [87]. These data indicate that circulating miRNAs need to be further studied in order to consider them useful biomarkers for monitoring dietary adherence in CD.

##### Mean Platelet Volume (MPV)

MPV indicates the average size of platelets. This is known by performing a blood test, and it is associated with heart disease, anemia and diabetes, among others. Several studies have recognized MPV as a biomarker of inflammation, in addition to observing its relationship to the severity of various diseases. In turn, other researchers have studied its potential role as a biomarker of dietary adherence in patients with celiac disease [4].

Specifically, MPV was measured in newly diagnosed celiac individuals and compared to healthy ones, with lower levels found in the latter group. Moreover, a significant decrease in MPV was observed in patients who strictly adhered to the GFD in comparison to those who did not [88,89]. Additionally, apart from higher MPV values, these patients showed villous atrophy in their biopsies; therefore, a relationship between this parameter and the inflammation of the intestine has been proposed [89].

##### High Molecular Group Box 1 (HMGB1)

HMGB1 is a protein found in both the cytoplasm and in the cell nucleus, released in response to inflammation, tissue damage or cell apoptosis. It has many biological functions; on the one hand, it has intracellular nuclear functions such as a structural role and gene transcription control, and, on the other hand, it has extracellular functions by triggering the immune response with the production of macrophages, NK cells and dendritic cells [8,90].

Clinical trials carried out in people with celiac disease have shown that their HMGB1 levels in plasma are significantly higher than those of healthy subjects [90]. The relationship of this biomarker with tTG-IgA has been also tested, and controversial outcomes have been obtained [90,91]. By contrast, it seems that the correlation of this biomarker with intestinal biopsies is clearer since significant differences have been observed in the HMGB1 levels of celiac patients with a Marsh grade of 3B-B1 and patients with a grade of 3C-B2, whereas no differences have been found between patients with a grade of 3A and patients with a grade of 3B-B1 [90]. Due to this correlation, HMGB1 has been proposed as a valid biomarker of enteropathy. It must be pointed out that the mentioned studies have only been performed on pediatric patients; therefore, more clinical trials are needed in adult patients to support the results observed in children.

##### Interleukines (ILs)

Interleukins are cytokines produced by cells of the immune system and are involved in the inflammation process. Considering their relationship with inflammation, their involvement in CD has been studied. Some cytokines have been analyzed in the diagnosis of CD because of their observed correlation with villous atrophy [4,5,92,93].

The presence of cytokines in the plasma of celiac patients treated with GFD and also in that of patients subjected to gluten challenges has been analyzed in different studies [92,93,94]. Results show that an increase in the levels of IL-2 (highest level), IL-6, IL-8, IL-10, IL-22 and IL-17A occurs after gluten ingestion and, moreover, the increase in different interleukins correlates with each other [93]. Other authors have also described higher expression in histological samples and plasma levels of IL-33 in celiac participants compared to healthy ones [95].

In view of all these results, it seems that the levels of some cytokines are affected in CD, with an increase observed after gluten intake. Even so, as only a few research works have been carried out to measure cytokine levels, more studies are needed to conclude whether they could be useful as biomarkers for the monitoring of CD.

##### Zonulin

Zonulin is a family of proteins involved in the tight junctions of the small intestine and, therefore, its permeability [96,97]. In CD, tight junctions fail, permeability increases and gluten peptides cross the epithelial barrier, triggering a response of the immune system [96]. Taking this into consideration, zonulin has been proposed as a biomarker of disrupted barrier function in humans to monitor CD and its follow-up [98].

Studies performed in children with CD have shown that zonulin serum levels significantly rise in the period previous to diagnosis and at diagnosis, demonstrating that this protein could be used as a biomarker for preclinical CD screening in at-risk children [96]. Drago et al. described how patients with CD exhibited an exaggerated, prolonged increase in small intestine permeability, and they observed that zonulin release in intestinal samples was of a significantly longer duration, persisting for 1 h or more in these patients, compared to healthy controls, whose luminal zonulin levels returned to baseline within 30 min [99].

The determination of this parameter has also been used for monitoring CD. In this sense, Duerksen et al. measured serum zonulin concentration and intestinal permeability (tested with lactulose/mannitol ratios) in adult celiac patients going on a GFD for longer than 1 year. The authors observed a clear correlation between intestinal permeability and zonulin levels. However, the complete normalization of these parameters after going on a GFD was only reached in a subgroup of patients, but not in the rest, probably due to continued gluten ingestion [100]. Studies performed in children have also demonstrated increased levels of this protein at diagnosis but significant reductions as a strict GFD is stablished [101].

##### Calprotectin

Calprotectin is a protein family mainly found within neutrophils and throughout the human body. The presence of calprotectin in feces is a consequence of neutrophil migration into the gastrointestinal tissue due to an inflammatory process. Therefore, fecal calprotectin is used as a biomarker in gastrointestinal inflammatory disorders [102]. Although it has been mainly used as a biomarker of inflammatory bowel disease (IBD), several authors have reported elevated calprotectin levels also at the onset of CD, especially when intestinal symptoms and significant histological changes are present [103,104]. However, other authors have exposed some discrepancies concerning the use of this protein in CD diagnosis, since they did not see a correlation between its concentration and the degree of histologic changes observed in biopsies [105].

The potential usefulness of this protein for CD follow-up needs to be studied more as well. Some authors observed reduced values in people with celiac disease going on a GFD [106], whereas in other studies, no statistically significant differences were detected between patients on a GFD and those recently diagnosed [101].

##### Lipocalin-2

Lipocalin-2, also referred to as neutrophil gelatinase-associated lipocalin, is a protein expressed by several cell types, including neutrophils and enterocytes, with several functions such as antibacterial, anti-inflammatory, and protection effects against cell and tissue stress [107]. In inflammatory diseases such as IBD, fecal lipocalin-2 concentration is elevated in patients with active disease and decreases with mucosal healing [108,109]; thus, some authors have proposed its utility for diagnosis and monitorization of other similar pathologies, such as CD.

However, the current bibliography describes controversial data in relation to this protein and its role in CD. For instance, Sutton et al. observed that lipocalin-2 is elevated in the stool but not in the plasma of patients with CD, which suggests its role only in local inflammatory response [106]. Regarding its levels in people going on a GFD, a study performed in children with celiac disease who were divided in two groups (one following a GFD and the other not following a GFD) observed no differences in plasma lipocalin between them [110]. These findings indicate that more studies are needed in order to include lipocalin-2 as a possible biomarker for the monitorization of CD.

##### Nitric Oxide

Nitric oxide (NO) is a radical gas produced by nitric oxide synthase whose production is elevated during inflammation. It has been assumed that in CD, gliadin toxicity causes oxidative stress with an increase in the concentration of reactive oxygen species and a decrease in antioxidant capacity. Due to this oxidative imbalance, the expression of nitric oxide synthase, as well as other pro-inflammatory cytokines, is activated, which in turn leads to an increased production of NO.

Studies have observed a higher activity of nitric oxide synthase enzyme and NO levels in patients with untreated CD and the normalization of them in patients with celiac disease on a GFD [111,112,113]. Moreover, some authors also described a statistically significant correlation between serum NO levels and the degree of histologic changes [113]. Taking this as a whole, it can be concluded that the current bibliography supports the measurement of NO serum levels (or, in general, measurements related to oxidative stress) as a useful biomarker for the monitorization of CD.

**Table 1:** Summary of biomarkers related to CD with a description of the observed changes in their levels in people with celiac disease before and after going on a GFD.

Biomarker	Sample	Function	Levels in People with CD before and after a GFD	References
I-FABPs	Blood	Biomarker of intestinal damage and GFD monitoring	Increased plasma levels in celiac patients due to enterocyte damage. Decreased after a GFD.	Adriaanse et al. (2017) [55] Vreugdenhil et al. (2011) [56] Adriaanse et al. (2013) [57] Adriaanse et al. (2016) [58]
CYP3A4	Blood (by administration of Simvastatin) Biopsy	Biomarker of intestinal damage	Decreased expression after gluten ingestion because it is expressed in the intestinal villi. Increased after a GFD.	Lang et al. (1996) [65] Johnson et al. (2001) [64] Moron et al. (2013) [66]
GIPs	Urine Stool	Biomarker of GFD follow-up	Increased levels in urine and feces in response to gluten ingestion.	Moreno et al. (2017) [68] Comino et al. (2019) [71] Comino et al. (2016) [72] Comino et al. (2012) [73]
Citrulline	Blood	Biomarker of intestinal damage	Decreased levels in people with CD, due to enteropathy. Increased after a GFD.	Ioannou et al. (2011) [80] Blasco-Alonso et al. (2011) [82] Crenn et al. (2003) [81] Singh et al. (2008) [83]
miRNA	Blood	Miscellaneous	Increased plasma levels of miR 192-5p, 215-5p, 125b-5p, 21, 155, 125 in celiac patients. Decreased plasma levels of miR-31 in celiac patients. No significant differences found between patients on a GFD and those with a GCD.	Felli et al. (2022) [85] Amr et al. (2019) [86] Bascuñán et al. (2020) [87]
MPV	Blood	Biomarker of intestinal inflammation and GFD monitoring	Increased plasma levels of MPV with increased intestinal atrophy in CD patients. Decreased after a GFD.	Gerceker et al. (2022) [89] Purnak et al. (2011) [88]
REG 1α	Blood	Biomarker of intestinal damage	Increased plasma levels in celiac disease patients. Decreased after a GFD.	Planas et al. (2011) [77]
HMBG1	Blood Stool	Biomarker of intestinal damage	Increased plasma and stool levels with increased intestinal atrophy in CD patients.	Manti et al. (2017) [90] Palone et al. (2018) [91]
ILs	Blood	Miscellaneous	Increased plasma levels of IL-2, 6, 8, 10, 17A, 22 and 33 in celiac patients.	Goel et al. (2020) [92] Goel et al. (2019) [93] Tye Din et al. (2020) [94] López Casado et al. (2017) [95]
Zonulin	Blood Stool	Biomarker of intestinal damage	Increased plasma levels in CD patients. Decreased after a GFD.	Da Fonte et al. (2024) [96] Rabiee et al. (2006) [98] Drago et al. (2006) [99] Duerksen et al. (2010) [100] Martínez Gallego et al. (2024) [101]
Calprotectin	Stool	Biomarker of intestinal inflammation	Increased levels at diagnosis.	Balamtekin et al. (2012) [103] Ertekin et al. (2010) [104] Sutton et al. (2024) [105] Oribe Bubica et al. (2021) [106] Martinez Gallego et al. (2024) [101]
Lipocalin-2	Blood Stool	Biomarker of intestinal inflammation	Increased levels in stool but not in plasma in CD patients.	Sutton et al. (2024) [105] Janas et al. (2016) [110]
Nitric oxide	Blood	Biomarker of oxidative stress and inflammation	Increased plasma levels in celiac patients. Decreased after a GFD.	Daniels et al. (2005) [111] Beckett et al. (1998) [112] Ertekin et al. (2005) [113]

### 3.2. Nutritional Perspective: Dietary Balance Achievement and Avoidance of Nutritional Deficiencies When Going on a GFD

Currently, the only effective treatment for CD is a lifelong GFD [1,2,3,5]. This diet must not only guarantee food safety by ensuring the total absence of gluten but also provide nutritional balance for the individual, fulfilling his or her energy and nutrient requirements. Regarding the nutritional profile, several studies have shown that the diet of celiac patients treated with a GFD is unbalanced [2,3,5,10]. This diet requires the use of GF cereals—corn, rice, sorghum, millet, teff—and pseudo-cereals—buckwheat, quinoa, amaranth, canihua—but also other foods that are naturally GF—potatoes, tapioca, nuts, oilseeds, legumes, fruits and vegetables—. Although theoretically simple, the GFD has many complexities, and it should not only be gluten-free but also balanced, covering total energy and nutritional requirements. Nevertheless, several studies have found imbalanced profiles of GFDs, characterized by low cereal, fruit and vegetable intake and excessive intake of meat and derivatives [12,98,114]. A deficiency of micronutrients such as vitamin D, iron and calcium has been observed in the adult population, and a deficiency of folate, zinc and magnesium has been observed among children. Regarding macronutrients, its consumption is characterized by low complex carbohydrate and fiber intake, in addition to high fat (especially saturated fats) and sugar intake [9,10,11,12]. This imbalance could be, at least in part, because CD patients on a GFD tend to consume specific processed GFPs, and these products have been shown to be poorer nutritionally than their gluten-containing homologues, which are often characterized for being high in saturated fat and salt and low in fiber [13,14,115].

Given this situation, it is strongly recommended to implement a personalized nutritional follow-up for celiac patients, led by dietitian nutritionists. In fact, authors working in this field have proposed a regular monitoring of dietary history, in addition to measuring serum antibodies and body composition as well as an examination of symptoms related to nutritional deficiencies, as a strategy for improving the nutritional status of celiac disease sufferers and for making GFDs more balanced [116,117]. In this sense, it is important to highlight that professionals working with this population have difficulties finding suitable and available electronic devices for an appropriate evaluation of a GFD and that they can usually only make approximations. To fill this gap, the Gluten3S research group designed the free open digital platform, *GlutenFreeDiet*, for the design and evaluation of GFDs (http://www.ehu.eus/dieta-singluten, accessed on 5 May 2024). This platform contains different sections that process clinical data of people with celiac disease, including anthropometric data, biochemical parameters, dietary habits and symptom presence. For dietary evaluation, apart from analyzing the nutritional composition of all conventional foods, this platform details the composition of more than 700 GF rendered foodstuffs, including breads, muffins, cereals, biscuits, etc. Therefore, this software allows dietitians to precisely measure a GFD’s energy content and nutrient distribution, in addition to the impact of specific GFPs on total energy intake [117,118].

### 3.3. Psychological Perspective: Psychological Assessment in Order to Avoid Mental Disorders

Regarding psychological aspects, individuals with CD may be at a higher risk for psychological disorders compared to healthy children and adults. CD is associated with an increased risk of depression, anxiety, eating disorders and autism spectrum disorders [119], contributing to a lower quality of life (QoL). Actually, people who suffer from CD report limitations when eating outside, constant worry about gluten, continuous planning, feeling different, emotional pressure or coping with symptoms. Right after diagnosis, many patients feel anger, fear, shame, rage and sadness [120], but after some time on a GFD, the situation might normalize, and they begin to feel control over the situation and feel good, their health-related QoL being improved [121,122]. However, as gluten avoidance is essential for people with CD, there is also concern that “extreme vigilance” to a strict GFD may increase symptoms such as anxiety, stress and fatigue and therefore reduce their QoL. It is important to consider the importance of promoting adherence to a diet together with emotional and social wellness and finding a balance. In team-based treatment focused on children, for instance, the active involvement of a psychologist plays a crucial role. Their work includes screening and intervention for psychological conditions, ensuring successful education of both the child and the family about this gastrointestinal condition and its management. Certainly, the impact of CD extends beyond the patient, and it affects the well-being of caregivers and the wider family. The family must also be considered when educating and supporting these patients [123]. Additionally, the psychologist must assist in coping with the challenges of diagnosis and managing symptoms while promoting adherence to the prescribed treatment. One of the key strategies highlighted in this collaborative approach is the provision of coping skills, such as social skills, acceptance and control. These tools serve as essential elements when trying to mitigate the impact of CD at any age. Moreover, the emphasis on social support contributes significantly to the development of awareness, fostering optimal long-term adherence to a GFD [122].

### 3.4. Social Perspective: Promotion of Celiac Integration in Society

CD has a significant impact on the QoL of people suffering from it, especially on a social level [15,124,125]. Numerous studies have highlighted the fact that individuals with CD frequently encounter feelings of being different and excluded. As explained above, this can be attributed to the difficulties they face when going out; they must be constantly vigilant about avoiding gluten, they are wary of the gluten-free designation of food on a menu, restaurant options are limited, sharing food is truly complicated, staff are sometimes dismissive or uninformed and they can become frustrated when they face accidental exposure. Travelling can also be especially challenging [17,125,126,127,128]. Furthermore, in situations such as when there is no GF food available if not ordered or when the only food available has gluten in it, people with CD must continuously communicate their condition and ask questions related to their diet. This draws attention to themselves, which is often unwanted [17,129].

Moreover, given that one of the most difficult aspects to cope with is eating outside, training people working in the catering industry and raising their awareness of the disease is of great necessity [130,131]. Several studies have shown that their knowledge of the disease is low [131,132,133,134,135], and due to the current “trend” of GFDs, the importance of the disease has been underestimated, and sufferers need to advocate for themselves even more [17]. Therefore, providing education to catering staff in this field could be of significant value in improving the situation and social inclusion of people with CD. In line with this, collective caterings should guarantee the availability of meals for those with celiac disease—in particular, in school canteens, rest homes and hospitals, as is actually possible in numerous countries [136].

On the other hand, in the case of families with a child diagnosed with CD, poor parental knowledge and attitudes toward the disease have been observed in some studies [137,138,139], and it has also been noted that the general population’s awareness of CD is low [132,140,141]. In order to promote the social inclusion of people with CD, it would be interesting to implement educational activities aimed at people close to those with CD and also at the general population. In a study conducted by Wolf et al., it was observed that for people with CD, the greatest source of relief for them was having supportive family and friends [17]. Nevertheless, if the general population also knew about the disease, were aware and familiar with the dietary needs people with celiac disease have and reacted supportively, social nonconformity could be reduced [130].

## 4. Conclusions

Following a strict GFD, the only current treatment for CD, can be difficult due to its restrictive profile and the need to avoid gluten contamination. Small ingestions of this protein can cause intestinal damage that can take a long time to recover from. Therefore, monitoring the treatment with a routine control of dietary transgressions and dietary counseling becomes mandatory. Traditional clinical methods to monitor the disease include invasive techniques such as intestinal biopsies. In this present review, a combination of different noninvasive methods were proposed, such as dietary questionnaires and the detection of biomarkers. Although data collected in questionnaires are important in order to detect possible gluten contamination sources and incorrect culinary practices, they can sometimes be subjective and inaccurate. For this reason, other more reliable, useful and objective measurements are proposed in combination with tools that truly reflect the patient’s condition, which are the recently described biomarkers.

Apart from its security, a GFD should ensure nutritional balance as well. Studies have demonstrated nutritional deficiencies among people with celiac disease on a GFD, which could compromise their nutritional status and health. Thus, nutritional assessment by professionals such as dietitians specializing in CD should be mandatory for these patients, and this requires tools that include both natural and specific gluten-free products. Thus, nutritional assessment is proposed as the second component in the follow-up of CD that should be considered.

Another matter of concern recently described for people with celiac disease is their mental health. The symptomatology and difficulties with strictly following the treatment can make these people feel stressed or even, on occasion, suffer from serious mental disorders such as anxiety and depression. Moreover, people with celiac disease feel misunderstood by society because of the lack of knowledge about the disease and GFDs. This feeling increases every time they must eat outside, where they must explain their condition and usually doubt the security of the meals they are offered. Thus, in order to avoid this situation, many people prefer to eat separately, excluded from the rest. Considering these problems, it is clear that psychological attention and social education must be focused on increasing knowledge about the disease and its treatment in general society, and these should be the third and the fourth necessary components included in the follow-up of CD.

Taking all of this information into account, it is clear that the wellness of people with celiac disease goes further than traditional clinical measurements and the set-up of a GFD. Once a diagnosis is established, celiac patients must face many difficulties when going on a GFD, which makes them have doubts and fears and feel disregarded and isolated. Therefore, a complete approach to the CD follow-up should be holistically established from four different perspectives: clinical, nutritional, psychological and social ones.
